# Temporal variability of *Loa loa* microfilaraemia

**DOI:** 10.1186/s13071-022-05612-0

**Published:** 2023-01-23

**Authors:** Jérémy T. Campillo, Marlhand C. Hemilembolo, Frédéric Louya, Paul Bikita, Sébastien D. S. Pion, Michel Boussinesq, François Missamou, Cédric B. Chesnais

**Affiliations:** 1 TransVIHMI, INSERM Unité 1175, Institut de Recherche pour le Développement (IRD), Université de Montpellier, Montpellier, France; 2 Programme National de Lutte contre l’Onchocercose, Direction de l’Épidémiologie et de la Lutte contre la Maladie, Ministère de la Santé et de la Population, Brazzaville, Republic of Congo

**Keywords:** *Loa loa*, Variability, Filariasis, Microfilaraemia

## Abstract

**Background:**

The diurnal periodicity of *Loa loa* microfilaraemia is well known but few studies have documented the short- and long-term stability of microfilarial density. It seems stable over time at the community level, but significant variations have been observed at the individual level.

**Methods:**

We assessed the temporal variability of *L. loa* microfilaraemia at 5-day, 1-month and 16-month intervals and analyzed the influence of sex, age, level of microfilaraemia, temperatures and time of sampling on this variability.

**Results:**

At the community level, *L. loa* microfilaraemia is very stable over time at 5-day, 1-month and 16-month intervals (Pearson correlation coefficients of 0.92, 0.91 and 0.78, respectively, all three with *P* < 0.001). However, some individuals had significant variations of up to ± 50% of their initial microfilaraemia at 5-day (33.0%), 1-month (36.5%) and 16-month (62.6%) intervals, even in individuals with an initial microfilaraemia density > 20,000 mf/ml (7.7, 23.1 and 41.4%, respectively, for 5 days, 1 month and 16 months). We do not highlight any external factors that have a major impact on this variability.

**Conclusion:**

Although at the community level, microfilaria density is very stable, we highlight some individuals with large variations in both the short and long term, which may have an important impact on onchocerciasis control campaigns and longitudinal studies evaluating the impact of an intervention on *L. loa* microfilaraemia.

**Graphical Abstract:**

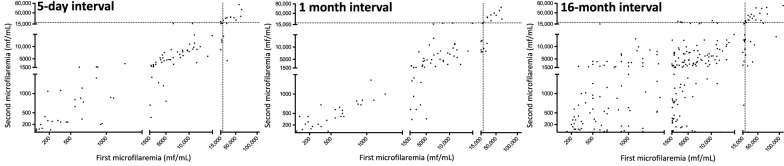

**Supplementary Information:**

The online version contains supplementary material available at 10.1186/s13071-022-05612-0.

## Background

Loaisis is a chronic infection due to the longevity of adults (up to 20 years). As a dioecious species, *Loa loa* produce embryos, called microfilariae (mf), that live in the bloodstream for about 6 months. Regardless of the short lifespan of mf, parasitological surveys have shown that the *L. loa* microfilarial density has high inter-individual variability (over-dispersion), but it is generally agreed that, beyond its diurnal periodicity, *L. loa* microfilarial density (MFD) of infected subjects left untreated remains stable over time (over several days, months or years). To the best of our knowledge, three studies have reported *L. loa* MFD measurements over consecutive days in the same untreated individuals (“short-term variability”). The first study was conducted in 1900 in five Nigerian prisoners whose MFD was measured 8–9 times at 3:00 p.m. within a 1-month interval, including 6 consecutive days. The authors considered that two of these five prisoners presented huge variations in MFD from day to day (varying between 160 and 1780 mf/ml at 21-day intervals and between 920 and 80 mf/ml at 6-day intervals) [1]. The second study was conducted in 1921 in a patient whose MFD was measured daily at noon for 16 consecutive days before starting a treatment course with antimonium tartaratum [2]. During the follow-up, the MFD fluctuated between 600 and 2200 microfilariae per ml of blood (mf/ml). In 1950, Kershaw also investigated the short-term variability of *L. loa* MFD in 59 Cameroonian prisoners who were examined daily at noon for 2–13 days. Among these subjects, 21 showed *L. loa* mf in at least one instance. Of these, Kershaw stated that 14 (66.7%) showed mf at each examination and 7 had intermittent microfilaraemia (at some time points, no mf was found). Logically, the subjects who were positive at all time points had higher MFD levels. In addition, Kershaw investigated changes in the MFD levels over a 4-day period in 193 persons living in a village in Nigeria. Of those, 43 showed *L. loa* mf in at least one instance and 38 (88.4%) were positive at all time points [3]. In these two populations, Kershaw did not explore quantitative variations in *L. loa* MFD; he only stated that “the numbers of microfilariae found varied widely.”

The first study assessing the long-term variability in *L. loa* MFD (i.e. over several months or years) was conducted in the Republic of the Congo [4]. A total of 192 individuals were screened by calibrated (40 µl) thick blood smears (TBS) in February 1985, and 171 and 66 of them were re-examined 2 months and 3 years later, respectively. No individual reported having taken any anti-filarial treatment during the interval. The arithmetic means of MFDs remained stable over time in the 42 microfilaremic subjects examined at 2-month intervals (3.3 mf/40 µl, then 3.2 mf/40 µl), and individual measurements showed a Pearson’s correlation coefficient *ρ* = 0.94 (*P* < 0.001). In the 28 microfilaremics reexamined after 3 years, the initial and final MFDs (3.7 and 3.8 mf/40 µl) were also highly correlated (*ρ* = 0.79, *P* < 0.001). A second study on the long-term variability of *L. loa* MFD was conducted in Cameroon [5]. A total of 667 individuals were examined every 2 months over a period of 1 year, including 195 microfilaremics sampled at least twice. Again, the prevalence of microfilaraemia and the MFD were very stable over time. In a third study, also conducted in Cameroon, 291 subjects excluded from ivermectin treatment because of contraindications (pregnancy, acute illness) or because of high *L. loa* MFD (> 20,000 mf/ml) associated with a possible risk of severe adverse event [6] were re-examined 18 months later [7]. Among those 173 who were microfilaremic at baseline, 94 (54.3%) remained in the same MFD category (1–100; 101–500, 501–2000, 2001–10,000, 10,001–20,000, > 20,000) 18 months later; however, 53 of 134 individuals (39.5%) excluded from ivermectin treatment because their MFD exceeded the risk threshold (20,000 mf/ml) had their MFD reduced below this value 18 months later. In a fourth study, changes in MFD levels were investigated over a 23-year interval (between 1993 and 2016) in 39 individuals [8]. The authors concluded that arithmetic mean MFD was similar between the two time points, though a slight increase occurred in 2016 (2280.5 mf/ml in 1993, 3080 mf/ml in 2016). None of these observational studies documenting the short- or long-term variability in *L. loa* individual MFD addressed the potential effects of intrinsic or extrinsic factors on MFD changes. In the present study, we assessed the effects of age, sex, and ambient temperatures on the day of sampling and time of blood sampling on the stability of MFD. Beyond its academic interest, this information might be relevant when preparing protocols for trials requiring follow-up of *L. loa* MFDs.

## Methods

**Fig. 1:**
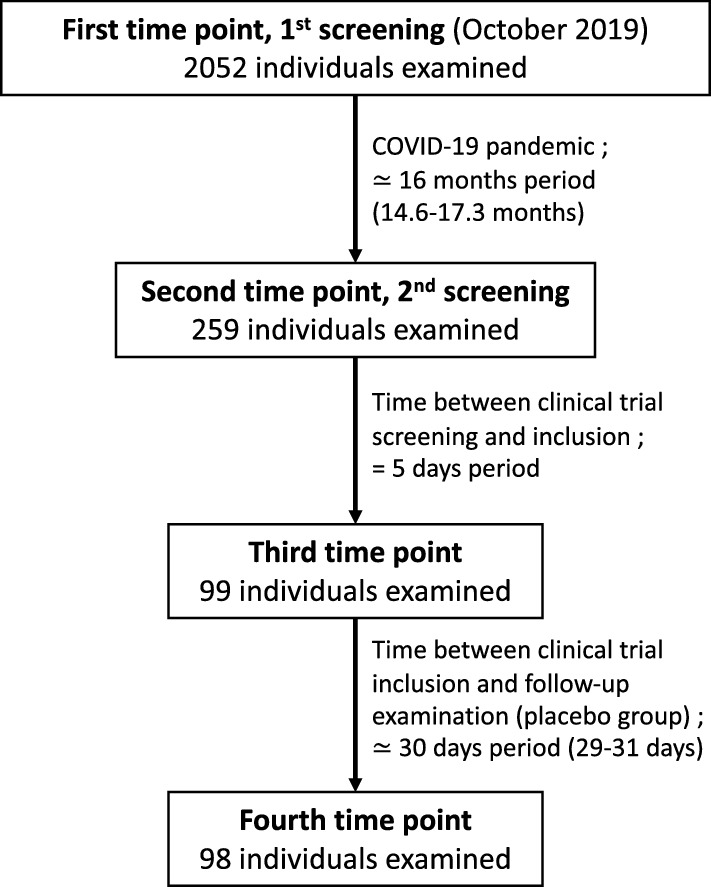
Flowchart of the study

Data were collected from individuals living in villages located near Sibiti, the capital town of the Lékoumou Division in the Republic of Congo. These individuals were examined as part of a placebo-controlled clinical trial evaluating the safety and effect of levamisole on *L. loa* MFD [9]. This trial consisted of measuring baseline participants’ MFD a few days before treatment and monitoring their MFD on Day 2 (D2), D7 and approximately D30 (from D29 to D31) after treatment with levamisole or placebo. Individuals were first examined in October 2019 to identify those meeting the inclusion criteria for the trial, which was supposed to start on March 2020. Due to the COVID-19 pandemic, the trial had to be postponed, and a second screening survey had to be organized between January and March 2021. Here, we analyzed MFD measured at a 5 day-interval (between D2 and D7) in the placebo group, at a 1-month interval (between D2 and D30) in the placebo group and at a 16-month interval (between the two screening surveys and prior to clinical trial inclusion; Fig. 1).

MFD were quantified using calibrated TBS. Blood was drawn by finger prick with a sterile lancet and collected in nonheparinized capillary tubes. A 50-μL volume of blood was spread on a slide, dried at room temperature, dehemoglobinized and stained with Giemsa within 24 h of collection. *Loa loa* mf were counted under a microscope using 100-fold magnification. Each slide was read by two microscopists who were blinded to donor identify and time point. The average ambient temperatures on each day of sampling were provided by the Sibiti weather station.

Pearson correlation coefficients between first MFD and second MFD were calculated. Variability measures were calculated as relative MFD differences between the initial assessment and subsequent follow-up assessments defined as follows: [(Second MFD − First MFD)/First MFD] × 100. The percentages of individuals for whom the MFD-relative difference (MFD-RD) was at least ± 10, ± 20, ± 30, ± 50 and ± 100% were calculated for the whole group and among each of the following categories of initial MFD: 1–500, 501–1000, 1001–2000, 2001–5000, 5001–10,000, 10,001–20,000 and > 20,000 mf/ml. Transition matrices were constructed to represent the evolution of MFD over time between MFD categories. As mentioned above, the threshold of 20,000 mf/ml had been used as a criterion to exclude individuals from ivermectin treatment as part of another study [10]. Thus, we analyzed particularly individuals whose MFD passed above or below this threshold between two time points.

For each of the follow-up intervals (5 days, 1 month and 16 months), we stratified the MFD-RD according to the first and third interquartile (ITQ) range. From this variable, we constructed three groups: (A) the 25% of individuals having the greatest decrease in MFD; (B) the 50% of individuals having the lowest change in MFD (reference); (C) the 25% of individuals having the greatest increase in MFD. Finally, we performed a multinomial regression model evaluating the influence of sex, age (categorized into 10-year interval), initial MFD (categorized to have a similar number of individuals in each category: 1–999, 1000–4999, 5000–11,999 and ≥ 12,000 mf/ml), ambient temperature and time of sampling for the probability to belong to group (A), (B) or (C). All analyses were performed using STATA software version 15.1.

The protocol of the trial (ClinicalTrials.gov Identifier: NCT04049630) during which the data analyzed in this article was collected was approved by the Committee on Ethics in Health Sciences Research of the Republic of Congo (no. 226/MRSIT/IRSSA/CERSSA), and an Administrative Authorization (no. 469/MSP/CAB/UCPP-19) was released by the Ministry of Health and Population of the Republic of Congo (MHPRC) [9]. Samples and data were collected by personnel for the MHPRC and before each step (screening surveys and inclusion in the clinical trial proper); each participant was given an information notice in French and in the local language (Lingala) and asked to sign an informed consent form (in both languages).

## Results

**Fig. 2:**
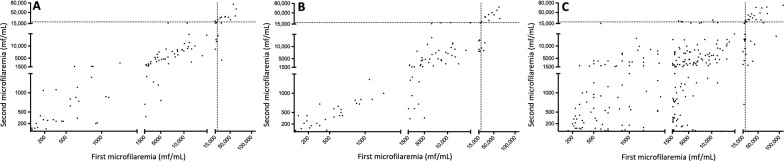
Correlation between initial and final microfilaraemia. **a** 5-day interval, **b** 1-month interval, **c** 16-month interval

Arithmetic mean MFDs decreased from 9541.0 to 8507.9 mf/ml at 5-day interval, from 9541.0 to 9281.4 mf/ml at 1-month interval and from 8528.3 to 6809.9 mf/ml at 16-month interval. Figure 2 shows, for each participant, their initial and latter MFD at 5-day, 1-month and 16-month intervals. The ρ coefficients between the initial and the latter MFD are 0.92 (*P* < 0.001), 0.91 (*P* < 0.001),and 0.78 (*P* < 0.001) at 5-day, 1-month and 16-month intervals, respectively. Table 1 shows the means, medians, quartiles, first and last percentiles, minima and maxima of the MFD-RD and MFD absolute difference for each of the follow-up periods.

**Table 1:** Main measures of microfilaraemia variability at 5-day, 1-month and 16-month intervals and variables of interest

	5-Day interval (*n* = 99)		1-Month interval (*n* = 98)		16-Month interval (*n* = 259)	
	Absolute (mf/ml)	Relative (%)	Absolute (mf/ml)	Relative (%)	Absolute (mf/ml)	Relative (%)
*Microfilaraemia difference*						
Mean ± SD	− 1033 ± 5550	+ 12.6 ± 82.6	− 1316 ± 5600	− 8.1 ± 82.4	− 1718 ± 9218	+ 42.5 ± 237.5
Median	− 160	− 5.5	− 170	− 23.6	− 600	− 29.9
10th percentile	− 5960	− 62.6	− 6400	− 70.1	− 7260	− 95.7
1st quartile	− 1400	− 27.5	− 2720	− 48.8	− 2980	− 71.0
3rd quartile	+ 540	+ 32.6	+ 100	+ 5.6	+ 760	+ 50.0
90th percentile	+ 2600	+ 100	+ 4820	+ 81.9	+ 5620	+ 248
Minimum	− 26,040	− 100	− 31,820	− 100	− 36,100	− 100
Maximum	+ 18,220	+ 489	+ 10,200	+ 600	+ 18,220	+ 768
*Time sampling difference*^*a*^						
Minimum	− 111 min		− 79 min		− 325 min	
Maximum	+ 61 min		+ 70 min		+ 225 min	
1st quartile	+ 0 min		− 2 min		− 115 min	
3rd quartile	− 2 min		+ 0 min		+ 43 min	
*Ambient temperature difference*^*a*^						
Minimum	− 3.1 °C		− 1.3 °C		− 3.5 °C	
Maximum	+ 2.9 °C		+ 5.0 °C		+ 5 °C	
1st quartile	− 3.1 °C		− 1.3 °C		− 0.3 °C	
3rd quartile	+ 0 °C		+ 0.5 °C		+ 1.9 °C	

*SD* standard deviation

^a^Second minus first time

At 5-day interval, only one subject became mf negative (his initial MFD was 60 mf/ml). At 1-month interval, three subjects became mf negative in MFD (their initial MFDs were 20, 20 and 60 mf/ml). Finally, at 16-month interval, 13 subjects became mf negative (4 with initial MFDs between 200 and 500 mf/ml, 4 with initial MFDs between 501 and 1000 mf/ml, 2 with initial MFDs between 4000 and 5000 mf/ml and 3 with initial MFDs of 12.120, 26.060 and 28.200 mf/ml).

**Fig. 3:**
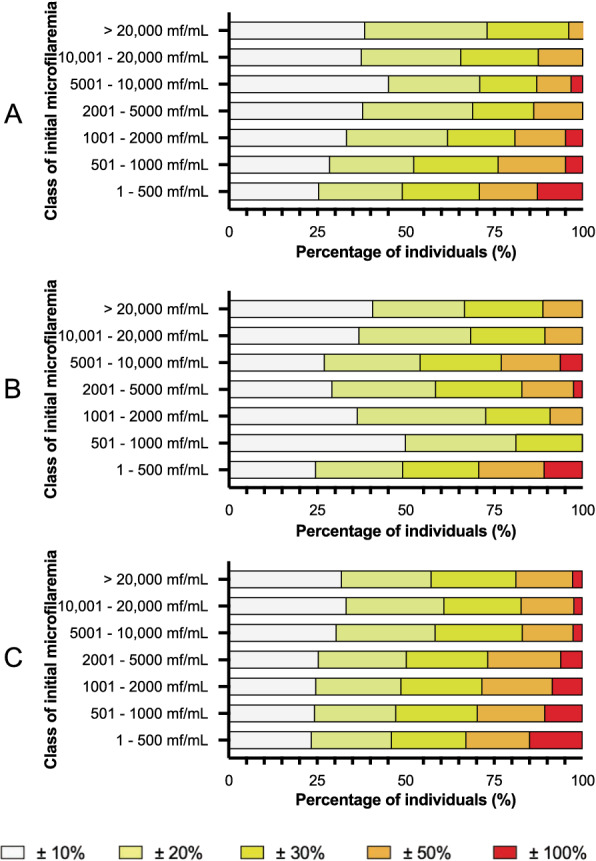
Variations in microfilaraemia by class of initial microfilaraemia at 5-day, 1-month and 16-month intervals. **a** 5-day interval, **b** 1-month interval, **c** 16-month interval

Figure 3 represents the proportions of individuals whose MFD varied by > 10, 20, 30, 50 and 100% from first MFD at 5-day, 1-month and 16-month intervals, respectively (data in Additional file 1: Table S1). A total of 58 of 99 (58.6%), 51 of 98 (52.0%) and 85 of 259 (32.8%) individuals remained in the same MFD category between the baseline assessment, and the measurement was made 5 days (Table 2), 1 month (Table 3) and 16 months (Table 4) after. When restricting the comparison at 16 months to those whose two samples were taken at the same time of the day ± 30 min, 17 of 58 (29.3%) remained in the same MFD category (Table 4). Among those subjects who had an MFD < 20,000 mf/ml at baseline, one (out of 99) exceeded this threshold 5 days after (increase from 15,020 to 22,920 mf/ml), none (out of 98) did so 1 month after and three (out of 259) did so 16 months after (from 2820 to 23,420, from 10,200 to 26,000 and from 3400 to 21,600 mf/ml). Conversely, three individuals (out of 13) changed from a MFD > 20,000 mf/ml to a lower MFD at 5-day interval; three (out of 13) did so at 1-month interval, and 12 (out of 29) did so at 16-month interval.

**Table 2:** *Loa loa* microfilaraemia transition matrix at 5-day interval

D0 (mf/ml)	D5 (mf/ml)							
	1–500	501–1000	1001–2000	2001–5000	5001–10,000	10,001–20,000	> 20,000	Total
1–500	16	0	2	0	0	0	0	18
501–1000	3	4	5	0	0	0	0	12
501–2000	1	3	1	3	0	0	0	8
2001–5000	0	0	3	7	4	0	0	14
5001–10,000	0	1	0	4	11	1	0	17
10,001–20,000	0	0	0	1	6	9	1	17
> 20,000	0	0	0	1	0	2	10	13
Total	20	8	11	37	21	12	11	99

Left column: result at Day 0; top row: result at Day 5

**Table 3:** *Loa loa* microfilaraemia transition matrix at 1-month interval

D0 (mf/ml)	D30 (mf/ml)							
	1–500	501–1000	1001–2000	2001–5000	5001–10,000	10,001–20,000	> 20,000	Total
1–500	17	1	0	0	0	0	0	18
501–1000	4	7	0	0	0	0	0	11
501–2000	1	3	2	2	0	0	0	8
2001–5000	0	3	5	4	2	0	0	14
5001–10,000	1	0	0	8	4	4	0	17
10,001–20,000	0	0	0	1	9	7	0	17
> 20,000	0	0	0	0	2	1	10	13
Total	23	14	7	15	17	12	10	98

Left column: result at Day 0; top row: result at Day 30)

**Table 4:** *Loa loa* microfilaraemia transition matrix at 16-month interval

2019 (mf/ml)	2021 (mf/ml)							
	0–500	501–1000	1001–2000	2001–5000	5001–10,000	10,001–20,000	> 20,000	Total
0–500	22 [2]	7 [1]	4 [2]	2 [2]	0 [0]	0 [0]	0 [0]	35 [7]
501–1000	17 [3]	6 [0]	6 [3]	6 [0]	2 [0]	1 [0]	0 [0]	38 [6]
501–2000	11 [2]	9 [3]	4 [1]	7 [2]	9 [0]	2 [1]	0 [0]	42 [9]
2001–5000	9 [1]	5 [0]	10 [2]	9 [4]	8 [2]	2 [1]	2 [0]	45 [10]
5001–10,000	2 [0]	1 [0]	2 [1]	16 [4]	11 [3]	5 [4]	0 [0]	37 [12]
10,001–20,000	1 [0]	1 [0]	1 [0]	6 [0]	7 [3]	16 [1]	1 [0]	33 [4]
> 20,000	2 [0]	0 [0]	0 [0]	2 [0]	2 [0]	6 [4]	17 [6]	29 [10]
Total	64 [8]	29 [4]	27 [9]	48 [12]	39 [8]	32 [11]	20 [6]	259 [58]

Left column: initial result; top row: final result

In brackets are represented individuals whose two samples were taken during the same time period (± 30 min)

At 5-day interval, multinomial regression model on MFD-RD changes (Additional file 1: Table S2) indicates that females were more likely to experience greatest MFD-RD increases [relative risk ratio (RRR) = 0.2; *P* = 0.050] than males. Individuals with the lowest initial MFDs (1–999 mf/ml) were more likely to experience the largest MFD-RD decreases (RRR = 16.9; *P* = 0.026) compared with individuals with high initial MFDs and individuals with the highest initial MFDs (≥ 12,000 mf/ml) were more likely to experience the largest MFD-RD increases (RRR = 11.2; *P* = 0.042) compared with individuals with lower initial MFDs. At 16-month interval, individuals with the highest initial MFDs (5000–11,999 and ≥ 12,000 mf/ml) were more likely to experience the largest MFD increases (RRR = 0.3; *P* = 0.022 and RRR = 0.1; *P* = 0.002, respectively) than individuals with low initial MFDs. Finally, outdoor temperature differences, difference in sampling time and age did not impact MFD-RD at 5-day, 1-month and 16-month intervals.

## Discussion

At 5-day, 1-month and 16-month intervals, participants’ mean MFDs appeared stable over time (highly significant ρ coefficients of 0.92, 0.91 and 0.78, respectively). These results are consistent with the literature. However, when looking at the individual level, larger variations than those reported in the literature were seen. More than 30% of the subjects had a ± 50% variation in their MFD-RDs at 5-day, 1-month and 16-month intervals. Although mainly present in subjects with low MFDs, which logically generate larger percentages of variation, we also found large variations of up to ± 50% in the high MFD categories (≥ 10,001 mf/ml). In addition, amazingly, three subjects whose initial MFD exceeded 10.000 mf/ml were found amicrofilaramic 16 months after. Although we cannot formally exclude anthelminthic intake, further studies could help to better understand this wide variability. This could be due to the physiological characteristics of the individuals, to lifestyle habits they share or to medicinal plants they may take.

Comparing relative differences is complicated with count data (as MFDs), because intuitively, large variations are more likely to occur when individual MFDs are small (50% relative increase is equivalent to increasing from 20 to 30 mf/ml as well as to increasing 2000–3000 mf/ml). However, the multinomial analysis by group included an adjustment of the initial MFD, which enabled to control for this factor when looking at other covariates. In addition, this analysis could highlight common characteristics within the population with the greatest decrease in MFD (group A) and within the population with the greatest increase in MFD (group C) compared to the subjects whose MFDs remained more stable (group B). We did not identify any external factors (temperature or sampling time) that had a significant impact on the variability of MFD using multinomial or linear regressions. However, our sample sizes were relatively small, especially for short-term variation, and extrinsic factors varied only slightly over the course of the study. Samples were taken at fairly regular times: for 5-day and 1-month interval, > 70% of samples had a time difference < 15 min. For 16-month interval, 40% of samples had a time difference < 1 h. Larger differences in sampling time would probably have had an impact on our results. Finally, daily temperatures varied only slightly over the course of the study. The MFD variations observed at long term (16-month interval) could also be due to other intrinsic factors (worm fecundity variation, deaths of adult words and/or microfilariae) or extrinsic factors, such as a temporary absence of some subjects from the villages during the season of high transmission (between November and May), that were not studied in this study [11, 12]. Entomological studies and biological investigations using non-human primate models of infections may help to answer these specific points.

During the trial, each patient had a participant card allowing us to identify him/her and thus limit errors in identity surveillance. In addition, each person was instructed not to self-medicate with an antihelminthic or to inform the team if they did so.

Only one (1%) and three individuals (1.2%) passed above the 20,000 mf/ml risk threshold within the 5-day and 16-month intervals, respectively. These results could have implications for future MDA campaigns, possibly meaning that, if a test-and-treat strategy is applied [10], it is essential to measure the *L. loa* MFD just before treatment. Moreover, we have shown that there is a significant variability at 5-day interval. Therefore, in the context of clinical trials, it seems essential to perform the TBS just before the administration of the treatment to be evaluated.

Our results have some limitations. Indeed, the variability observed may be partly due to the variability induced by technicians’ accuracy when reading the TBS with a microscope. In addition, although each participant was asked if he/she had taken a treatment since the last sampling, a memory bias could have led to inexact answers.

## Conclusion

Our results show a strong stability of MFD over time at the community level. However, we also showed a significant variability in some individuals. This variability could potentially influence the results of longitudinal studies such as clinical trials with the absolute or relative difference in *L. loa* MFD as a decision criterion. We also draw attention to the existence of a few individuals who exceeded the 20,000 mf/ml threshold, which may have implications for MDA programs.

## Supplementary Information

**Additional file 1: Table S1.** Variations in microfilaraemia by class of initial microfilaraemia at 5-day, 1-month and 16-month intervals. **Table S2.** Multinomial regression model comparing the 25% of individuals with the greatest decrease in initial microfilaraemia and the 25% of individuals with the greatest increase in initial microfilaraemia to the individuals between these two extremes.

## Data Availability

Data supporting the conclusions of this article are included within the article. The datasets used and/or analyzed during the present study are available from the corresponding author upon reasonable request.
