# Supplementary material for: Temporal variability of Loa loa microfilaraemia
Source: Parasit Vectors. 2023 Jan 23;16:23. doi: 10.1186/s13071-022-05612-0 (PMC9869825; doi:10.1186/s13071-022-05612-0)
Supplement: Supplementary file 1 — Additional file 1: Table S1. Variations in microfilaraemia by class of initial microfilaraemia at 5-day, 1-month and 16-month intervals. Table S2. Multinomial regression model comparing the 25% of individuals with the greatest decrease in initial microfilaraemia and the 25% of individuals with the greatest increase in initial microfilaraemia to the individuals between these two extremes. [file 13071_2022_5612_MOESM1_ESM.docx]

Supplementary material. **Temporal variability of *Loa loa* microfilaremia**

|  | **5-day interval** | | | | | | **1-month interval** | | | | | | **16-month interval** | | | | | |  |
| --- | --- | --- | --- | --- | --- | --- | --- | --- | --- | --- | --- | --- | --- | --- | --- | --- | --- | --- | --- |
|  | **MFD variation < 1st ITQ** | | | **MFD variation > 3rd ITQ** | | | **MFD variation > 1st ITQ** | | | **MFD variation > 3rd ITQ** | | | **MFD variation > 1st ITQ** | | | **MFD variation > 3rd ITQ** | | | |
|  | RRR | 95% CI | *P* | RRR | 95% CI | *P* | RRR | 95% CI | *P* | RRR | 95% CI | *P* | RRR | 95% CI | *P* | RRR | 95% CI | *P* | |
| **Sex** | | | | | | | | | | | | | | | | | | |  |
| Women | Ref. |  |  | Ref. |  |  | Ref. |  |  | Ref. |  |  | Ref. |  |  | Ref. |  |  | |
| Men | 0.7 | 0.1 – 4.0 | 0.657 | 0.2 | 0.1 – 1.0 | 0.050 | 0.4 | 0.1 – 1.5 | 0.190 | 1.1 | 0.3 – 4.5 | 0.838 | 1.0 | 0.5 – 2.0 | 0.940 | 2.2 | 1.0 – 4.8 | 0.057 | |
| **Age** | | | | | | | | | | | | | | | | | | |  |
| 18–28 years | 6.2 | 0.2 – 175.3 | 0.281 | 5.6 | 0.2 – 150.3 | 0.306 | 6.1 | 0.7 – 52.9 | 0.103 | 1.4 | 0.2 – 10.5 | 0.754 | 0.7 | 0.2 – 2.5 | 0.608 | 0.9 | 0.3 – 2.9 | 0.898 | |
| 29–38 years | Ref. |  |  | Ref. |  |  | Ref. |  |  | Ref. |  |  | Ref. |  |  | Ref. |  |  | |
| 39–48 years | 0.1 | 0.1 – 2.4 | 0.182 | 0.6 | 0.1 – 7.1 | 0.715 | 4.3 | 0.5 – 35.6 | 0.179 | 2.5 | 0.4 – 14.3 | 0.317 | 0.7 | 0.2 – 2.3 | 0.585 | 0.9 | 0.3 – 2.8 | 0.821 | |
| 49–58 years | 1.6 | 0.1 – 16.3 | 0.711 | 0.6 | 0.1 – 6.4 | 0.646 | 3.0 | 0.5 – 20.6 | 0.259 | 1.2 | 0.2 – 6.2 | 0.860 | 1.1 | 0.4 – 3.1 | 0.850 | 0.6 | 0.2 – 1.6 | 0.286 | |
| 59 years and more | 0.3 | 0.1 – 4.6 | 0.397 | 0.5 | 0.1 – 6.0 | 0.583 | 5.6 | 0.7 – 41.9 | 0.090 | 3.0 | 0.5 – 18.1 | 0.220 | 1.3 | 0.4 – 3.9 | 0.670 | 0.9 | 0.3 – 2.8 | 0.841 | |
| **Initial microfilaremia (mf/mL)** | | | | | | | | | | | | | | | | | | |  |
| 1–999 | 16.9 | 1.4 – 204.0 | 0.026 | 7.0 | 0.7 – 65.7 | 0.089 | 1.0 | 0.26 –4.0 | 0.990 | 1.5 | 0.3 – 6.3 | 0.580 | 1.3 | 0.6 – 3.0 | 0.559 | 1.3 | 0.6 – 2.8 | 0.568 | |
| 1000–4999 | Ref. |  |  | Ref. |  |  | Ref. |  |  | Ref. |  |  | Ref. |  |  | Ref. |  |  | |
| 5000–11,999 | 4.6 | 0.6 – 35.3 | 0.138 | 0.6 | 0.1 – 4.6 | 0.666 | 0.2 | 0.1 – 1.4 | 0.113 | 0.5 | 0.1 – 2.7 | 0.426 | 0.5 | 0.2 – 1.2 | 0.131 | 0.3 | 0.1 – 0.8 | 0.015 | |
| 12,000 and more | 2.6 | 0.2 – 29.6 | 0.434 | 11.2 | 1.1 – 115.3 | 0.042 | 0.4 | 0.1 – 2.2 | 0.316 | 2.0 | 0.5 – 8.6 | 0.333 | 0.5 | 0.2 – 1.2 | 0.109 | 0.1 | 0.0 – 0.3 | 0.002 | |
| **Difference in sampling time** | | | | | | | | | | | | | | | | | | |  |
| < 15 min. | Ref. |  |  | Ref. |  |  | Ref. |  |  | Ref. |  |  | Ref. |  |  | Ref. |  |  | |
| ≥ 15 min. | 1.5 | 0.1 – 15.3 | 0.720 | 2.3 | 0.2 – 22.0 | 0.467 | 3.0 | 0.7 – 13.0 | 0.139 | 0.8 | 0.1 – 1.5 | 0.790 | 2.3 | 0.7 – 7.3 | 0.151 | 2.4 | 0.8 – 7.7 | 0.133 | |
| **Outdoor temperature difference (Second temperature - First temperature)** | | | | | | | | | | | | | | | | | | |  |
| < – 2°C | 0.3 | 0.1 – 2.0 | 0.235 | 3.6 | 0.6 – 22.4 | 0.173 | Not included* | | | | | | 1.3 | 0.4 – 3.7 | 0.666 | 1.8 | 0.7 – 4.6 | 0.244 | |
| – 2°C to + 2°C | Ref. |  |  | Ref. |  |  |  |  |  |  |  |  | Ref. |  |  | Ref. |  |  | |
| > + 2°C | 5.8 | 0.1 – 9.8 | 0.648 | 4.5 | 0.2 – 86.4 | 0.319 |  |  |  |  |  |  | 1.2 | 0.5 – 2.6 | 0.661 | 0.6 | 0.3 – 1.6 | 0.350 | |

**Supplementary material 1. Multinomial regression model comparing the 25% of individuals with the greatest decrease in initial microfilaremia and the 25% of individuals with the greatest increase in initial microfilaremia to the individuals between these two extremes.**

RRR, Relative risk ratios; Ref., Reference; ITQ, Interquartile; MFD, Microfilarial densities; C, Celsius; CI, Confidence intervals

* Not included because not enough variations in outdoor temperatures

The class of reference is the individuals for whom their initial MFD varied between the 1st and the 3rd ITQ.

|  | **5-day interval (n = 40)** | | | **1-month interval (n = 30)** | | | **16-month interval (n = 89)** | | |
| --- | --- | --- | --- | --- | --- | --- | --- | --- | --- |
|  | Coeff. | 95% CI | *P* | Coeff. | 95% CI | *P* | Coeff. | 95% CI | *P* |
| **Sex** | | | | | | | | | |
| Women | + 1971 | + 619 – + 3323 | 0.004 | + 411 | - 2656 – + 3480 | 0.792 | Ref. |  |  |
| Men | Ref. |  |  | Ref. |  |  | + 1150 | - 1254 – + 3556 | 0.349 |
| **Age** | | | | | | | | | |
| 18–28 years | Ref. |  |  | + 2632 | - 555 – + 5819 | 0.105 | + 1741 | - 1048 – + 4531 | 0.221 |
| 29–38 years | + 2984 | + 905 – + 5062 | 0.005 | + 1109 | - 2859 – + 5078 | 0.584 | + 1216 | - 1509 – + 3942 | 0.382 |
| 39–48 years | + 3036 | + 1327 – + 4745 | < 0.001 | + 3390 | + 547 – + 6233 | 0.019 | + 1094 | - 1725 – + 3914 | 0.447 |
| 49–58 years | + 2064 | + 486 – + 3631 | 0.010 | Ref. |  |  | Ref. |  |  |
| 59 years and more | + 2409 | + 225 – + 4592 | 0.031 | + 1585 | - 1627 – + 4798 | 0.333 | + 3724 | + 866 – + 6582 | 0.011 |
| **Initial microfilaremia (mf/mL)** | | | | | | | | | |
| 1–999 | + 970 | - 2199 – + 4140 | 0.549 | Ref. |  |  | Ref. |  |  |
| 1000–4999 | + 1134 | - 2529 – + 4797 | 0.544 | + 1515 | - 1659 – + 4690 | 0.350 | + 3639 | + 1530 – + 5749 | 0.001 |
| 5000–11,999 | Ref. |  |  | + 3611 | - 54 – + 7278 | 0.053 | + 5971 | + 2834 – + 9108 | < 0.001 |
| 12,000 and more | + 7349 | + 4093 – + 10605 | < 0.001 | + 4386 | + 1195 – + 7577 | 0.007 | + 5701 | + 2181 – + 9221 | 0.002 |
| **Sampling time frame*** | | | | | | | | | |
| Same time frame | Ref. |  |  | + 2139 | - 1399 – + 5678 | 0.236 | Ref. |  |  |
| Different time frame | + 342 | - 1640 – + 2325 | 0.735 | Ref. |  |  | + 83 | - 2194 – + 2360 | 0.943 |
| **Difference in outdoor temperature**** | | | | | | | | | |
| < – 2°C | + 174 | - 1148 – + 1496 | 0.796 | Not included *** | | | + 2344 | - 943 – + 5632 | 0.163 |
| – 2°C to + 2°C | Ref. |  |  |  |  |  | + 1913 | - 754 – + 4579 | 0.160 |
| > + 2°C | - 898 | - 3509 – + 1712 | 0.500 |  |  |  | Ref. |  |  |
| **Random effect on the village of residence** | ICC = 89.0% (P = 0.004) | | | Not significative | | | Not significative | | |

Supplementary material 2. Linear regression of the absolute difference in microfilarial densities at 5-days, 1-month and 16-months among individuals who experienced an increase in their microfilaremia

* For 5-days and 1-month: samples are considered to be in the same time frame if it was taken within 15 minutes of each other. For 16-months interval: samples are considered to be in the same time frame if it was taken within 30 minutes of each other

** For temperature differences, understand second temperature – first temperature.

*** All individuals are in the same category

|  | **5-day interval (n = 55)** | | | **1-month interval (n = 65)** | | | **16-month interval (n = 154)** | | |
| --- | --- | --- | --- | --- | --- | --- | --- | --- | --- |
|  | Coeff. | 95% CI | *P* | Coeff. | 95% CI | *P* | Coeff. | 95% CI | *P* |
| **Sex** | | | | | | | | | |
| Women | Ref. |  |  | - 1525 | - 3844 – + 794 | 0.197 | Ref. |  |  |
| Men | - 549 | - 4152 – + 1952 | 0.480 | Ref. |  |  | - 249 | - 2319 – + 1822 | 0.814 |
| **Age** | | | | | | | | | |
| 18–28 years | - 549 | - 5997 – + 4899 | 0.843 | Ref. |  |  | - 597 | - 4017 – + 2822 | 0.732 |
| 29–38 years | - 3095 | - 7712 – + 1522 | 0.189 | - 1528 | - 5094 – + 2039 | 0.401 | - 1278 | - 4656 – + 2100 | 0.458 |
| 39–48 years | - 113 | - 4268 – + 4042 | 0.957 | - 3380 | - 6898 – + 139 | 0.060 | Ref. |  |  |
| 49–58 years | - 238 | - 4063 – + 3586 | 0.903 | - 1406 | - 4456 – + 1644 | 0.366 | - 855 | - 3479 – + 1768 | 0.523 |
| 59 years and more | Ref. |  |  | - 2421 | - 5968 – + 1126 | 0.181 | - 2385 | - 5562 – + 791 | 0.141 |
| **Initial microfilaremia (mf/mL)** | | | | | | | | | |
| 1–999 | - 66 | - 4522 – + 4390 | 0.977 | Ref. |  |  | Ref. |  |  |
| 1000–4999 | Ref. |  |  | - 1867 | - 4372 – + 647 | 0.144 | - 998 | - 3845 – + 1849 | 0.492 |
| 5000–11,999 | - 2748 | - 5931 – + 435 | 0.091 | - 4635 | - 4635 – - 1701 | 0.002 | - 3834 | - 7191 – - 477 | 0.025 |
| 12,000 and more | - 8427 | - 12985 – - 3870 | < 0.001 | - 6185 | - 10077 – - 2293 | 0.002 | - 12,239 | - 15550 – - 8928 | < 0.001 |
| **Sampling time frame*** | | | | | | | | | |
| Same time frame | Ref. |  |  | Ref. |  |  | Ref. |  |  |
| Different time frame | - 3604 | - 7350 – + 140 | 0.059 | - 1193 | - 3927 – + 1540 | 0.392 | - 53 | - 2483 – + 2377 | 0.966 |
| **Difference in outdoor temperature**** | | | | | | | | | |
| < – 2°C | - 1625 | - 7242 – + 3991 | 0.571 | Empty*** | | | - 974 | - 5933 – + 3985 | 0.700 |
| – 2°C to + 2°C | - 1232 | - 6101 – + 3637 | 0.620 | Ref. |  |  | - 501 | - 3847 – + 2845 | 0.769 |
| > + 2°C | Ref. |  |  | - 2088 | - 8047 – + 3870 | 0.492 | Ref. |  |  |
| **Random effect on the village of residence** | Not significative | | | Not significative | | | ICC = 80,7% (P < 0.0001) | | |

Supplementary material 3. Linear regression of the absolute difference in microfilarial densities at 5-days, 1-month and 16-months among individuals who experienced a decrease in their microfilaremia

* For 5-day and 1-month: samples are considered to be in the same time frame if it was taken within 15 minutes of each other. For 16-months interval: samples are considered to be in the same time frame if it was taken within 30 minutes of each other

** For temperature differences, understand second temperature – first temperature.

*** No individuals in this category
